# Risk Stratification Tools in Acute Heart Failure and Their Roles in Personalized Follow-Up

**DOI:** 10.3390/jcm14248937

**Published:** 2025-12-18

**Authors:** Vittoria Rizzello, Samuela Carigi, Renata De Maria, Maria Denitza Tinti, Raul Limonta, Francesco Orso, Matteo Bianco, Luisa De Gennaro, Maria Vittoria Matassini, Paolo Manca, Concetta Di Nora, Alessandro Navazio, Giovanna Geraci, Furio Colivicchi, Claudio Bilato, Federico Nardi, Massimo Grimaldi, Fabrizio Oliva

**Affiliations:** 1 Heart Failure Working Group, National Association of Hospital Cardiologists (ANMCO), 50121 Florence, Italyconcetta.dinora@asufc.sanita.fvg.it (C.D.N.); 2 Cardiology Department, A.O. San Giovanni Addolorata, 00184 Rome, Italy; 3 Cardiology Unit, Ospedale Infermi di Rimini, AUSL Della Romagna, 47923 Rimini, Italy; 4 Cardiology Unit, A.O. San Camillo Forlanini, 00152 Rome, Italy; 5 Department of Medicine and Surgery, University of Milano-Bicocca, 20126 Milan, Italy; 6 ANMCO Study Center, Fondazione per il Tuo Cuore, 50121 Florence, Italy; 7 Cardiology Department, A.O.U. San Luigi Gonzaga, 10043 Orbassano, Italy; 8 Cardiology Unit, San Paolo Hospital, 70123 Bari, Italy; 9 Cardiology-CCU Department, A.O.U. Delle Marche, 60126 Ancona, Italy; 10 Cardiology, Mediterranean Institute for Transplants and Highly Specialized Therapies (ISMETT IRCCS), 90127 Palermo, Italy; 11 Cardiac Surgery Department, Azienda Sanitaria Universitaria Friuli Centrale, 33100 Udine, Italy; 12 Hospital Cardiology Department, Arcispedale Santa Maria Nuova, Azienda USL of Reggio Emilia—IRCCS, 42122 Reggio Emilia, Italy; alessandro.navazio@ausl.re.it; 13 Cardiology-CCU Unit, Sant’Antonio Abate Hospital, 91016 Trapani, Italy; 14 Clinical and Rehabilitative Cardiology Department, San Filippo Neri Hospital, 00135 Rome, Italy; furio.colivicchi@gmail.com; 15 Cardiology Unit, Ospedale dell’Ovest Vicentino, Azienda ULSS 8 Berica, 36100 Vicenza, Italy; claudio.bilato@aulss8.veneto.it; 16 Cardiology Department, Ospedale Santo Spirito, 15033 Casale Monferrato, Italy; 17 Cardiology Unit, Miulli Hospital of Acquaviva Delle Fonti, 70021 Bari, Italy; 18 National Association of Hospital Cardiologists (ANMCO), 50121 Firenze, Italy; 19 Fondazione per il Tuo Cuore-Heart Care Foundation, 50121 Firenze, Italy; 20 Cardiology Unit, Niguarda Hospital, 20162 Milan, Italy; fabrizio.oliva@ospedaleniguarda.it

**Keywords:** heart failure, follow-up, risk stratification

## Abstract

Heart failure (HF) is a condition with a high clinical and healthcare burden. In Europe, the current incidence is approximately 5 per 1000 persons per year in the adult population, with a prevalence of 1–2%. Prognosis remains poor, with persistently high rates of mortality and hospitalization. The period immediately following hospital discharge is known as the “vulnerable phase,” marked by a heightened incidence of clinical events, including a 30% chance of rehospitalization and a 10% mortality risk. Effective patient management during this time is crucial and should incorporate a risk stratification process, which is vital for designing a personalized follow-up plan and ensuring optimal resource utilization. This review aims to outline the available tools for prognostic risk stratification and propose a structured follow-up model applicable at discharge from an HF hospitalization in patients with HF with reduced ejection fraction and in patients with mildly reduced/preserved ejection fraction.

## 1. Introduction

Heart failure (HF) is a condition with a significant clinical and healthcare impact [1]. The prognosis remains poor, as HF is characterized by high rates of hospitalization and mortality. These outcomes worsen progressively across different phenotypes: HF with preserved ejection fraction (HFpEF ≥ 50%), mildly reduced ejection fraction (HFmrEF, 41–49%), and reduced ejection fraction (HFrEF, <40%) [2].

In patients with HFrEF, clinical guidelines prioritize the rapid and, whenever possible, simultaneous initiation of the four pillars of guideline-directed medical therapy (GDMT): beta-blockers (BBs), angiotensin receptor–neprilysin inhibitors (ARNIs) or angiotensin-converting enzyme inhibitors/angiotensin receptor blockers (ACEi/ARBs), mineralocorticoid receptor antagonists (MRAs), and sodium–glucose cotransporter 2 inhibitors (SGLT2is) [3,4]. These therapies offer additive and early prognostic benefits. For patients with HFmrEF or HFpEF, SGLT2 inhibitors currently represent the only class I recommended pharmacologic treatment. In patients with worsening heart failure (WHF), vericiguat may be considered as an additional therapeutic option [3,4].

Hospitalization for HF (HFH) is a pivotal moment in the natural course of HF, increasing the risk of subsequent rehospitalization and mortality, but it also offers a valuable opportunity to initiate and titrate GDMT. The “vulnerable period” early post-discharge (typically within 1–3 months) carries a 30% risk of rehospitalization and a 10% risk of mortality [5,6]. Thus, treatment optimization and structured, close follow-up during this window are critical, since they affect the subsequent clinical trajectory, which may fluctuate between improvement, stability, or further deterioration. In the STRONG-HF trial, simultaneous in-hospital initiation during HFH of GDMT, followed by rapid up-titration, has been shown to be safe, well-tolerated, and effective across HF phenotypes [7]. However, although strongly recommended by international guidelines, the replicability of the STRONG-HF titration strategy in clinical settings is limited by scant healthcare resources and higher representation of elderly patients in the real world (i.e., one third of the population of the Italian BRING-UP 3 registry versus less than one fifth of the STRONG-HF population) [8,9].

Therefore, effective follow-up planning must include accurate risk stratification and targeted care strategies. Risk stratification should be actively implemented across various care settings, including Cardiology, Internal Medicine, and Emergency Departments, as well as specialized HF outpatient clinics and community-based healthcare services. Moreover, given the chronic and complex nature of HF, the development of integrated care models based on multidisciplinary teams (MDTs)—including physicians, nurses, and pharmacists—coordinated by a case manager to ensure continuity and personalization of care, is essential.

In this review, we will summarize the main tools for risk stratification and propose a personalized, “risk-stratified” follow-up model applicable at discharge from an HFH, specifically in patients with HFrEF and HFmrEF/HFpEF. Risk stratification tools were

## 2. Tools for Prognostic Risk Stratification

In order to provide a comprehensive review of all potentially useful tools for risk stratification, we performed a search in PubMed of articles published in English in the last 25 years regarding risk stratification in patients with HFrEF and HFmrEF/HFpEF. The keywords included: HF and risk stratification, prognosis prediction, assessment of congestion, echocardiography, natriuretic peptides (NPs), biomarkers, risk scores, frailty, comorbidities, advanced HF (AdvHF), implanted devices, artificial intelligence (AI). These variables were chosen based on their established prognostic role and/or on guidelines endorsement as well as clinical relevance.

### 2.1. Assessment of Residual Congestion

Congestion is defined as the presence of signs and symptoms of extracellular fluid overload due to elevated filling pressures. Over 80% of patients hospitalized for acute HF present signs of congestion, and up to 50% of them are discharged with some degree of subclinical congestion. This condition significantly increases the risk of early rehospitalization and six-month mortality and remains common even during the chronic phases of HF, negatively impacting prognosis [10,11].

Subclinical congestion requires a multiparametric assessment that combines various diagnostic modalities [12,13].

- **Clinical signs and symptoms** have low sensitivity for detecting residual or subclinical congestion. To enhance diagnostic accuracy, several clinical scores have been developed, though these are not routinely implemented in everyday clinical practice [12].
- **Lung ultrasound (LUS)** evaluates pulmonary congestion through quantification of comet-tail artifacts (B-lines): the number of B-lines correlates directly with congestion severity and is associated with a poorer prognosis. In a meta-analysis of 13 studies on acute HF, the presence of more than 15 B-lines at discharge was associated with a five-fold increased risk of rehospitalization and death [14]. In outpatient settings, having more than 3 B-lines identified patients at a four-fold increased risk [15].
- **Inferior vena cava (IVC) ultrasound** can evaluate persistent subclinical venous congestion through measurements of IVC diameter and its respiratory variation (optimal threshold values: ≤21 mm diameter with >50% collapsibility). For a more comprehensive assessment, a multiorgan ultrasound approach known as venous extended ultrasound (VExUS) has been proposed, which includes evaluation of the IVC, hepatic veins, portal vein, and renal vein [16].
  Further echocardiographic parameters to assess congestion include measurements of pulmonary and left ventricular filling pressures [17]. The presence of post-capillary or combined pre- and post-capillary pulmonary hypertension is associated with poor prognosis. Evaluation of right ventricular function also has prognostic value: specifically, the TAPSE/PASP ratio (tricuspid annular plane systolic excursion/systolic pulmonary artery pressure), with a cut-off value of 0.36, serves as a negative outcome marker [18].
  Another critical component in the evaluation of congestion is the measurement of NPs, which will be discussed in the next section.

Remote and home-based monitoring of congestion is also becoming increasingly feasible. Implantable devices equipped with multisensor software offer promising solutions. A systematic review and meta-analysis of eight randomized trials (4347 patients) demonstrated that these home-monitoring strategies are more effective than standard monitoring in reducing the composite endpoint of death and HF rehospitalization—primarily driven by reduced readmissions [19]. The CardioMEMS HF System, an implantable sensor placed in a branch of the left pulmonary artery, measures pulmonary pressure and transmits data to an external device for clinical review and remote management. A meta-analysis of three studies revealed a significant reduction in hospitalizations/urgent visits and all-cause mortality in HF patients associated with CardioMEMS use [20]. Nevertheless, patient selection criteria for this technology remain to be clearly defined.

In clinical practice, a comprehensive evaluation of congestion status—both at the time of discharge and during follow-up—should integrate clinical signs and symptoms, NP levels, and ultrasound findings from echocardiography, LUS, and VExUS [21,22,23,24,25,26] (Table 1). Because this multiparametric integration may be challenging due to technical limitations, operator variability, and differences in NP thresholds, efforts should be made to develop specialized skills and standardize the process of congestion evaluation as much as possible. Indexes of congestion from implanted devices should also be considered. We acknowledge that availability of technologies such as VExUS, ultrasound, or device-based software may vary across settings and therefore a protocol developed according to local facilities should be applied to assess congestion.

### 2.2. Risk Stratification by Disease Stage

The risk of death and rehospitalization in HF is closely associated with disease stage. A large systematic review including 862,046 patients evaluated 30-day and one-year all-cause mortality and HFH across different clinical stages. While outcomes at 30 days were relatively similar among groups, one-year event rates increased substantially with disease progression: from chronic stable HF (8.47%), to WHF (26.84%), and finally to AdvHF (29.74%), with de novo HF patients presenting a 21.15% incidence of negative outcomes [27].

WHF represents a phase of clinical deterioration in patients with previously stable chronic HF, typically requiring hospitalization and/or intensification of diuretic therapy [28]. Identification of WHF should prompt more aggressive management, including reassessment of GDMT, implementation of vericiguat, consideration of device-based interventions, valvular correction or coronary revascularization, and intensified follow-up.

In addition, timely recognition of AdvHF remains critical to avoid delayed referrals that may jeopardize the eligibility and effectiveness of advanced interventions. Risk stratification models can assist in identifying patients with AdvHF. A predicted one-year survival ≤ 80%, as estimated by established tools such as the Meta-Analysis Global Group in Chronic Heart Failure (MAGGIC) score, the Seattle Heart Failure Model (SHFM), or the Metabolic Exercise Cardiac Kidney Index (MECKI) score, is indicative of severe disease and may warrant referral for AdvHF therapies [29,30,31]. To facilitate early referral, the European Society of Cardiology (ESC) recommends the I-NEED-HELP acronym, which encompasses the following indicators of disease severity:

- **I**: inotropes;
- **N**: NYHA class III–IV or elevated natriuretic peptides;
- **E**: end-organ dysfunction;
- **E**: implantable cardioverter-defibrillator (ICD) shocks;
- **D**: decreased ejection fraction;
- **H**: hospitalization;
- **E**: edema or escalating diuretic requirement;
- **L**: low blood pressure;
- **P**: poor response to prognostic medical therapy.

Although the list was originally designed as a referral guide, a growing number of positive I-NEED-HELP criteria has been associated with progressively worse outcomes. Independent predictors of one-year mortality or re-hospitalization for HF include inotrope dependence, NYHA functional class III–IV, persistently elevated NT-proBNP levels, end-organ dysfunction, multiple hospital admissions, persistent congestion despite diuretic escalation, and hypotension [32].

Finally, cardiopulmonary exercise testing (CPET) is a well-validated prognostic tool in patients with AdvHF, particularly in those being evaluated for heart transplantation. CPET demonstrates prognostic value across all HF phenotypes. A peak oxygen consumption (pVO_2_) threshold of 14 mL/kg/min is commonly used to identify high-risk patients. Recently, a stratification into four categories of risk based on pVO_2_ values has been proposed: >20 mL/kg/min, 16–20 mL/kg/min, 10–16 mL/kg/min, and <10 mL/kg/min. Other major prognostic indices at CPET include the ventilatory efficiency slope (VE/VCO_2_) and the presence of exercise oscillatory ventilation. A VE/VCO_2_ slope > 34–36 is strongly associated with increased risk of adverse outcomes [33,34].

### 2.3. Biomarkers

A variety of biomarkers—reflecting different pathophysiological mechanisms involved in HF progression, including neurohormonal activation, myocardial injury and remodeling, inflammation, oxidative stress, and multiorgan damage—have demonstrated clinical value in HF risk stratification [35].

Among all biomarkers, the strongest prognostic evidence pertains to NPs. Multiple studies have shown that, in patients hospitalized for acute HF, elevated NP levels at admission are associated with increased in-hospital, short-term, and long-term mortality, with incremental prognostic value over traditional risk indices [35]. Moreover, the change in NP levels at discharge compared with admission serves as a surrogate marker of decongestion: a reduction of less than 30% from baseline is associated with poorer prognosis [5,36].

Persistently elevated NP levels during follow-up are linked to unfavorable outcomes in both HFrEF and HFpEF populations and are included among the criteria for identifying patients with AdvHF [37,38]. For individual patients, knowledge of the “dry” NP value—i.e., the level when the patient is fully decongested—is important. An increase of more than 50% from this dry value may indicate elevated filling pressures and relapsed congestion [13]. In the STRONG-HF trial, an increase of 10% in NP levels was used as a cut-off value indicative of early congestion, warranting temporary withholding of beta-blocker up-titration [7]. When interpreting natriuretic peptide (NP) levels, clinicians should recognize that several underlying conditions can influence NP concentrations. Specifically, kidney disease and atrial fibrillation tend to result in higher NP levels, while obesity is associated with lower NP levels. Although the value of an NP-guided therapeutic strategy remains uncertain—since no mortality benefit compared with standard care was demonstrated among 894 HFrEF patients in the GUIDE-IT trial—in clinical practice, serial NP measurements can nonetheless provide substantial clinical utility [39].

### 2.4. Assessment of Frailty and Comorbidities

The prognosis of patients with HF is not only driven by their cardiovascular disease but is also significantly affected by frailty and comorbidities.

Frailty is clinically defined as “a multidimensional dynamic state, independent of age, that makes the individual with HF more vulnerable to the effects of stressors” such as acute illness, hospitalization, or medical procedures [40]. This increased vulnerability is due to reduced physiological reserves, often amplified by muscle wasting, sarcopenia, and cardiac cachexia [41].

Frailty is common in HF, affecting up to 50% of patients depending on the population studied and the assessment tools used, and has been shown to increase the risk of all-cause mortality and hospitalization by over 40% [41,42,43]. Despite its clear clinical significance, frailty has often been omitted from conventional cardiovascular risk prediction models, and only recently has its assessment gained attention in clinical research and official guidelines [44,45,46].

Several qualitative and quantitative tools have been proposed to capture various aspects of health—physical function, sarcopenia, disability, comorbidity burden, cognitive status, and psychological health—all of which are often compromised in HF [47]. Among the various assessment metrics, gait speed has emerged as a reliable single-item marker of frailty. Reduced gait speed is a known predictor of negative outcomes in older adults, including falls, functional decline, hospitalization, and mortality. Furthermore, its inclusion in established risk models, such as the Cardiac and Comorbid Conditions Heart Failure (3C-HF) score, has enhanced the ability to predict all-cause mortality and HF-related hospitalizations [40,48].

Recently, Vitale et al. proposed the Heart Failure Frailty Score (HFFS) and its simplified version (sHFFS) to quantify frailty, with the latter appearing more applicable in clinical practice (approximately five minutes to administer) [40]. In brief, the sHFFS—developed through a Delphi consensus—includes evaluation across four domains: clinical (cardiovascular and non-cardiovascular comorbidities, unintentional weight loss, falls), functional (activities of daily living, mobility, 30 s chair stand test), psycho-cognitive (dementia, depression), and social (living arrangement, support). The ability of the sHFFS to stratify prognosis in patients with HF remains to be evaluated in future studies.

The prognostic impact of cardiovascular and non-cardiovascular comorbidities—which represent an important determinant of frailty—has been widely demonstrated in HF patients. A recent meta-analysis including 39 studies identified 10 comorbid conditions (such as diabetes, chronic obstructive pulmonary disease, chronic kidney disease, atrial fibrillation, ischemic heart disease, stroke, anemia, cancer, dementia, and depression) that are associated with adverse prognostic outcomes, including all-cause mortality, HF readmission, and all-cause readmission [49].

### 2.5. From Traditional Scores to Artificial Intelligence

Over the past 30 years, numerous risk scores have been developed to predict mortality and/or rehospitalization in patients with both acute and chronic HF. These scores have been derived from randomized controlled trials and observational cohorts, serving as valuable tools for tailoring follow-up strategies to individual risk profiles. However, the performance of risk scores often varies depending on the derivation cohort and the variables included.

An ideal HF risk model would:

- Reflect a broad, real-world HF population;
- Include relevant comorbidities;
- Encompass the full spectrum of left ventricular ejection fraction (LVEF);
- Integrate NPs, functional status, and echocardiographic parameters;
- Be regularly updated to reflect evolving therapies;
- Be simple to use and widely adopted in clinical practice [50].

Despite these desirable characteristics, the variables used across published scores remain heterogeneous, ranging from clinical to biological, functional, and imaging data. No single model consistently outperforms the others. In general, most proposed scores exhibit good discrimination but variable calibration, with some models underestimating the risk (e.g., the Seattle Heart Failure Score) and others overestimating it (e.g., the MAGGIC score) [51,52].

A meta-analysis involving 494,156 patients and 11 different scoring systems found that models designed for acute HF demonstrated excellent predictive accuracy for short-term mortality. In contrast, scores developed for chronic HF showed lower performance for medium- and long-term outcomes [53]. Other studies have confirmed that 1-year predictions tend to outperform those for longer-term follow-up [52]. Notably, scores predicting only mortality tend to be more accurate than those predicting both mortality and hospitalization, as the latter are influenced by external factors such as healthcare system dynamics and patient adherence [50].

A major limitation of most current tools is their inadequate performance in HFpEF, where risk stratification remains particularly challenging [54].

The advent of artificial intelligence (AI) offers new possibilities for overcoming the limitations of traditional risk scores and represents a promising approach for risk stratification at discharge and during chronic follow-up. Compared with classical models, AI offers several advantages:

- The ability to handle complex, multidimensional interactions between variables;
- Personalized risk prediction rather than population-level estimations;
- Greater adaptability to diverse patient populations and contemporary cohorts;
- Potential for integration into electronic health records, thereby reducing clinician workload and enabling real-time decision support [55].

Multiple studies have validated AI’s utility in this context:

- Golas et al. applied an AI model to retrospective data from 11,000 patients and found it outperformed traditional models in predicting 30-day hospital readmissions [56].
- Kwon et al. developed an AI system that successfully predicted in-hospital, 12-month, and 36-month mortality, surpassing the predictive power of conventional scores such as MAGGIC [57].
- Shah et al. employed machine learning techniques to identify HFpEF phenotypes with significantly different risks of mortality and hospitalization, recognizing three distinct clusters with unique outcomes [58].

Nevertheless, several challenges remain:

- Many AI models demonstrate only modest performance gains over traditional approaches;
- Data quality and consistency across diverse clinical settings is not always available, as datasets often vary in completeness, standardization, and representation;
- Classic issues such as missing data, limited generalizability, and competing risks persist;
- Most models rely on static data snapshots, whereas incorporating longitudinal trends could enhance predictive accuracy;
- The inclusion of a large number of variables can compromise clinical interpretability;
- Key prognostic data types—such as hemodynamic parameters, clinical narratives, imaging, and omics—are often underutilized in current AI applications

Still, ongoing advancements in AI and machine learning are expected to address these limitations. Integration of deep learning techniques, natural language processing for unstructured EHR data, and federated learning across institutions may further enhance AI’s performance and applicability in real-world HF management. However, clinical adoption can be slow, influenced by workflow integration issues, regulatory considerations, and the need for clinician trust in new tools.

## 3. Tailored Follow-Up Strategies

Implementing a structured follow-up model for patients with HF, as recommended by current guidelines, is often not feasible in routine clinical practice. Real-world data indicate that only 50–60% of patients during the “vulnerable phase” receive a follow-up visit within 14 days by a general practitioner, and merely 20–25% are seen by a cardiologist. As a result, GDMT prescriptions remain suboptimal in both completeness and achievement of target doses [59].

Accurate risk stratification, both in the early post-acute phase and throughout the chronic course of the disease, can support the design of tailored and sustainable follow-up pathways. However, among the various available stratification tools, risk scores are often difficult to apply, as required variables are not always available, and integration with other prognostic predictors or modern therapeutic options is not standardized. Furthermore, risk scores typically do not account for multidisciplinary management, telemedicine, or hospital–community integration. Consequently, despite the availability of online calculators, fewer than 1% of patients are stratified using a risk score during clinical evaluation [51].

More immediately applicable tools for risk-based patient stratification include:

- Biomarkers, particularly BNP/NT-proBNP;
- Clinical and echocardiographic assessment of residual congestion;
- Identification of clinical features indicative of AdvHF or WHF.

The use of a formal risk score may still be considered when the required variables are readily available. Among existing models, the MAGGIC score appears to be the most applicable, owing to its good discrimination and calibration performance (C-statistic: 0.74; Observed–Expected Ratio: 1.08) and the widespread availability of its component variables, which encompass several comorbidities [60]. Moreover, simple assessment tools such as the gait-speed test or the sHFFS may be useful for quantifying frailty and improving overall risk stratification. This is particularly relevant because frail patients require personalized follow-up with slower titration of medication, close monitoring of possible side effects and multidisciplinary consultation for appropriate care.

Based on these considerations, and acknowledging the heterogeneity of HF populations, two distinct follow-up scenarios can be defined after an acute event, guided by risk stratification: HFrEF and HFmrEF/HFpEF. In all settings, the case manager plays a pivotal role in ensuring timely access to care, integrating the efforts of multiple healthcare professionals, patients, and caregivers, and ensuring a seamless continuum of care across hospital and community environments.

(1) **HFrEF**

Patients hospitalized with acute HFrEF should undergo early risk stratification. High-risk features include:

- Presence of AdHF or WHF criteria;
- Clinical, echocardiographic, or biomarker evidence of residual congestion;
- Inadequate reduction in NP levels during hospitalization;
- Presence of multiple comorbidities and frailty.

In the absence of these risk factors, the patient may be considered at lower risk.

When I-NEED-HELP criteria for AdvHF are met, timely referral to an advanced HF centre is essential to prevent missing the therapeutic window for advanced interventions. This also allows for the development of shared care plans, including scheduled diuretic infusions or intermittent inotropic therapy.

Importantly, since only a small proportion of AdvHF patients will eventually access advanced therapies, early integration of palliative care is crucial during this vulnerable phase.

All HFrEF patients require timely GDMT initiation and up-titration. High-risk patients should receive close follow-up by a cardiologist—ideally with a first visit within 14 days and multiple evaluations in the following 6–9 weeks. For patients with frailty and multiple comorbidities an integrated multidisciplinary pathway should be available. Conversely, lower-risk patients may be followed primarily by nurses through in-person, telemedicine, or phone visits, with guided GDMT titration and medical reassessment at 3 months, or earlier if clinical warning signs emerge. We acknowledge that facilities such as nurse-led titration, telemonitoring access and device-based care might not be globally available and this may justify a less frequent follow-up in lower-risk patients.

The 3-month follow-up visit is essential to reassess risk, determine the patient’s clinical trajectory (e.g., improvement, stabilization, or WHF), and evaluate candidacy for additional interventions such as device therapy or valvular procedures.

**Figure 1:**
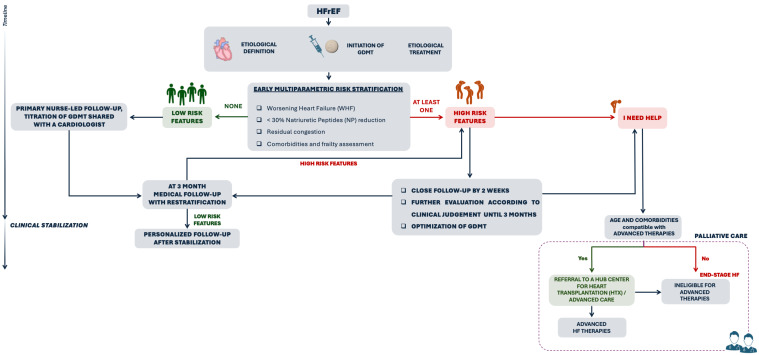
Personalized follow-up plan in HFrEF. Post-discharge follow-up plan is guided by multiparametric risk stratification. In the presence of high-risk features, such as AdvHF or WHF, residual congestion, insufficient reduction in NP levels or multiple comorbidities and frailty, a close (within 2 weeks) and intensive follow-up strategy is appropriate. In patients with I NEED HELP criteria, referral to an advanced HF centre is essential. Patients without high-risk features can be considered at low risk and should undergo GDMT titration by a nursing-led program shared with a cardiologist. At three months medical re-evaluation is needed, both in low and high-risk patients, to define patient’s trajectory and consider device implantation. Personalized follow-up should continue up to clinical stabilization (at least 6 months).

Based on this updated risk profile, a personalized follow-up plan should be established, covering at least the following 6 months, or until the patient achieves sustained clinical stabilization (Figure 1).

(2) **HFmrEF and HFpEF**

In patients with HFmrEF and HFpEF, risk stratification is primarily driven by the underlying aetiology, which must be thoroughly investigated given its implications for disease-specific management. Once reversible or specific causes are excluded, treatment with SGLT2 inhibitors and, based on the recent results of the FINEARTS-HF trial, finerenone should be initiated [61].

Although follow-up for HFmrEF and HFpEF is generally less intensive than that for HFrEF, the presence of frailty, multiple comorbidities, and the persistent risk of rehospitalization often complicate clinical management. In cases where congestion is not fully resolved at discharge, close monitoring is warranted until complete decongestion is achieved.

**Figure 2:**
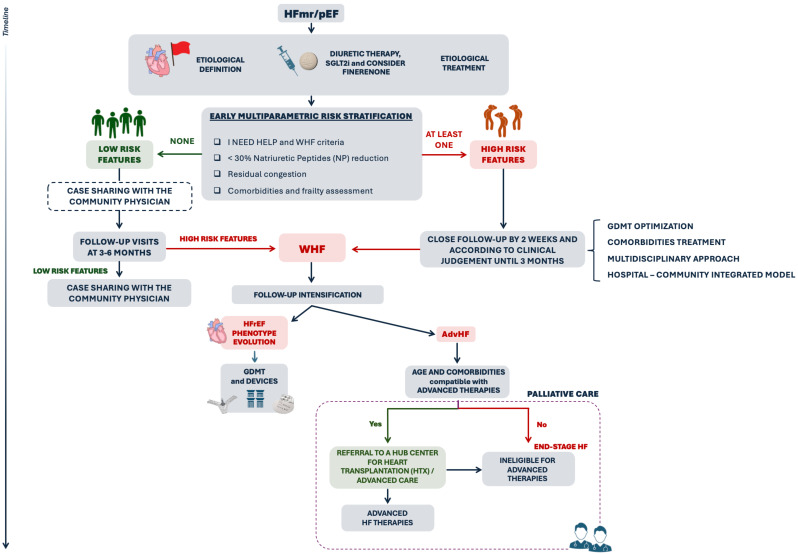
Personalized follow-up plan in HFmrEF and HFpEF. Multiparametric risk stratification guides the follow-up plan. In the presence of high-risk features, such as AdvHF or WHF, residual congestion, insufficient reduction in NP levels or multiple comorbidities and frailty, a more intensive follow-up strategy is warranted. This includes close clinical re-evaluation within 2 weeks and thereafter up to 3 months as needed. Patients without high-risk features can be considered at low risk. These individuals can be managed collaboratively with the community physician in the early post-discharge period, with further assessment by a cardiologist scheduled between 3–6 months. Regardless of the risk level, a multidisciplinary approach and a hospital-community integrated model should be applied to ensure comprehensive management of comorbidities. Should a patient develop WHF with progression to HFrEF phenotype, management should follow the pathway outlined in Figure 1. In presence of AdvHF, referral to an advanced HF centre is essential.

Given the central role of comorbidities in the pathophysiology of HFmrEF and HFpEF, their proactive and integrated management is essential. This should ideally occur at the community level, with coordinated input from general practitioners, geriatricians, and cardiologists, operating within a multidisciplinary, hospital–community integrated care model (Figure 2) [62].

Patients with HFmrEF and HFpEF may also progress to WHF or AdHF. In such scenarios—particularly given the high prevalence of frailty in this population—multidisciplinary management, frequent evaluations in cardiology day hospitals, and the early involvement of palliative care teams may provide significant clinical benefit.

## 4. Conclusions

HF follows a natural course characterized by alternating phases of clinical stability and acute decompensation. Effective long-term management requires a structured follow-up strategy, implemented through a multidisciplinary care team.

For optimal use of healthcare resources, management of HF patients should be guided by multiparametric risk stratification. High-risk patients should be prioritized for intensive, complex therapeutic programs, including frequent clinical assessments and advanced interventions. Conversely, for lower-risk individuals, standardized management protocols with more extended follow-up intervals may be sufficient and cost-effective.

Looking ahead, the integration of AI into routine HF management may refine the complex process of dynamic risk stratification across all stages of the disease, in order to support personalized care planning and improve cost-effective resource allocation.

## Figures and Tables

**Table 1:** Decongestion targets in patients with Heart Failure.

Congestion Parameter	Target	Reference
Clinical Parameters		
**Physical Examination**	- No signs of HF	Salah et al. Heart 2014 [21]
**NYHA Class**	- <II	
Biomarkers		
**NT-proBNP**	- >30% reduction at discharge <1500 pg/mL at discharge - No significant increase (>50%) at FU	McQuade et al. 2017 [26] Salah et al. Heart 2014 [21] Kociol et al. Circ Heart Fail 2011 [23]
**BNP**	- <250 pg/mL at discharge - No significant increase (>50%) at FU	McQuade et al. 2017 [26]
Imaging		
**VExUS**	**VCI** - maximum diameter < 2.1 cm - Collapsibility index > 50% **Hepatic veins:** - S > D **Portal and renal veins**: - non-pulsatile flow	Goonewardena et al. JACC Img 2008 [22] Stansen J, et al. JACC Cardiovasc Imaging. 2023 [16]
**Lung Ultrasound**	- <15 B-lines at discharge - <3 B-lines in outpatient setting	Platz et al. Eur J HF 2017 [15]
**ECHOCARDIOGRAPHY** - **Transmitral Doppler and TDI** - **TRV** - **sTDI** - **TAPSE** - **TAPSE/PAPs**	- E/E’ < 10 or ΔE/E’ > 5 - E/A ratio < 2 - DT > 130 ms - <2.8 m/s - >9.5 cm/s - >18 mm - >0.36	Lancellotti P et al. Eur HJ-CVI 2017 [24] Humbert et al. Eur Heart J 2022 [25] Guazzi et al. Am J Phisiol Heart Circ Phisiol 2017 [18]

BNP: Brain Natriuretic Peptide; DT: E-wave Deceleration Time; HF: Heart Failure; IVC: Inferior Vena Cava; NT-proBNP: N-terminal pro-Brain Natriuretic Peptide; NYHA: New York Heart Association, PAPs: systolic pulmonary artery pressure; sTDI: right ventricle’s S wave Tissue Doppler Imaging velocity; TAPSE: tricuspid annular plane systolic excursion; TDI: Tissue Doppler Imaging; TRV: tricuspid regurgitation velocity; VExUS: Venous Excess Ultrasonography Score.

## Abbreviations

The following abbreviations are used in this manuscript:

HF	Heart failure	
HFrEF	Heart failure with reduced ejection fraction	
HFmrEF	Heart failure with mildly reduced ejection fraction	
HFpEF	Heart failure with preserved ejection fraction	
GDMT	Guideline-directed medical therapy	
BBs	Beta-blockers	
ARNIs	Angiotensin receptor–neprilysin inhibitors	
ACEi/ARB	Angiotensin-converting enzyme inhibitors/angiotensin receptor blockers	
MRAs	Mineralocorticoid receptor antagonists	
SGLT2is	Sodium–glucose cotransporter 2 inhibitors	
WHF	Worsening Heart Failure	
AdvHF	Advanced Heart failure	
HHF	Hospitalization for Heart failure	
MDTs	Multidisciplinary teams	
LUS	Lung ultrasound	
IVC	Inferior vena cava	
VExUS	Venous extended ultrasound	
TAPSE/PASP	Tricuspid annular plane systolic excursion/systolic pulmonary artery pressure	
NPs	Natriuretic peptides	
NYHA	New York Heart Association	
BNP	Brain Natriuretic Peptide	
DT	E-wave Deceleration Time	
NT-proBNP	N-terminal pro-Brain Natriuretic Peptide	
sTDI	Right ventricle’s S wave Tissue Doppler Imaging velocity	
TDI	Tissue Doppler Imaging	
TRV	Tricuspid regurgitation velocity	
MAGGIC	Meta-Analysis Global Group in Chronic Heart Failure score	
SHFM	Seattle Heart Failure Model	
MECKI	Metabolic Exercise Cardiac Kidney Index	
I-NEED-HELP	I = inotropes; N: NYHA class III–IV or elevated natriuretic peptides; E = end-organ dysfunction; E = implantable cardioverter-defibrillator (ICD) shocks; D = decreased ejection fraction; H = hospitalization; E = edema or escalating diuretic requirement; L = low blood pressure; P: poor response to prognostic medical therapy	
CPET	Cardiopulmonary exercise testing	
pVO_2_	Peak oxygen consumption	
VE/VCO2	Ventilatory efficiency slope	
3C-HF	Cardiac and Comorbid Conditions Heart Failure	
HFFS	Heart Failure Frailty Score	
sHFFS	Simplified-Heart Failure Frailty Score	
AI	Artificial intelligence	
